# Tumour infiltrating lymphocytes correlate with improved survival in patients with oesophageal adenocarcinoma

**DOI:** 10.1007/s00262-016-1826-5

**Published:** 2016-03-28

**Authors:** Fergus Noble, Toby Mellows, Leo H. McCormick Matthews, Adrian C. Bateman, Scott Harris, Timothy J. Underwood, James P. Byrne, Ian S. Bailey, Donna M. Sharland, Jamie J. Kelly, John N. Primrose, Surinder S. Sahota, Andrew R. Bateman, Gareth J. Thomas, Christian H. Ottensmeier

**Affiliations:** 1 Cancer Sciences Unit, Faculty of Medicine, University of Southampton, Somers Cancer Research Building (MP824), Southampton General Hospital, Tremona Road, Southampton, UK; 2 Department of Cellular Pathology, University Hospital Southampton NHS Foundation Trust, Southampton, UK; 3 Department of Surgery, University Hospital Southampton NHS Foundation Trust, Southampton, UK; 4 Cancer Care, University Hospital Southampton NHS Foundation Trust, Southampton, UK; 5 Public Health Sciences and Medical Statistics, Faculty of Medicine, University of Southampton, Southampton, UK

**Keywords:** Cytotoxic T lymphocyte, Immune response, Immunotherapy, Oesophageal adenocarcinoma, Regulatory T cells, Tumour regression

## Abstract

**Background:**

Oesophageal adenocarcinoma (OAC) is increasingly common in the west, and survival remains poor at 10–15 % at 5 years. Immune responses are increasingly implicated as a determining factor of tumour progression. The ability of lymphocytes to recognise tumour antigens provides a mechanism for a host immune attack against cancer providing a potential treatment strategy.

**Materials and Methods:**

Tumour infiltrating lymphocytes (TILs: CD3+, CD4+, CD8+ and FOXp3+) were assessed by immunohistochemistry using tissue microarrays in a contemporary and homogeneous cohort of OAC patients (*n* = 128) undergoing curative treatment.

**Results:**

Multivariate analysis identified three independent prognostic factors for improved cancer-specific survival (CSS): increased CD8+ TILs (*p* = 0.003), completeness of resection (*p* < 0.0001) and lower pathological N stage (*p* < 0.0001). Independent prognostic factors for favourable disease-free survival included surgery-only treatment (*p* = 0.015), completeness of resection (*p* = 0.001), increased CD8+ TILs (*p* < 0.0001) and reduced pathological N stage (*p* < 0.0001). Higher levels of TILs in the pathological specimen were associated with significant pathological response to neoadjuvant chemotherapy (NAC). On multivariate analysis increased levels of CD4+ (*p* = 0.017) and CD8+ TILs (*p* = 0.005) were associated with significant local tumour regression and lymph node downstaging, respectively.

**Discussion:**

Our results establish an association of TILs and survival in OAC, as seen in other solid tumours, and identify particular TIL subsets that are present at higher levels in patients who responded to NAC compared to non-responders. These findings highlight potential therapeutic strategies in EAC based on utilising the host immunological response and highlight the immune responses biomarker potential.

**Electronic supplementary material:**

The online version of this article (doi:10.1007/s00262-016-1826-5) contains supplementary material, which is available to authorized users.

## Introduction

Virchow first described the association of lymphocyte infiltration with solid tumours in 1863 [1]. Immune responses against malignant cells, whether systemic or within the tumour microenvironment, are increasingly implicated as a determining factor in tumour progression [2]. The most comprehensive studies, to establish this hallmark of cancer [3], have been in colorectal tumours [4]. Specific tumour infiltrating lymphocyte (TIL) subsets communicate and function to affect tumour growth, and the balance of these effects leads to either tumour regression or tumour promotion. An anti-tumour effect is mediated by the combination of cancer cell lysis and the production of cytotoxic cytokines, supported by cluster of differentiation (CD)4+ T helper cells. In contrast, T regulatory cells, Forkhead box P3+ (FOXp3+) T cells, act to suppress the immune response of other cells and so affect the inflammatory process indirectly [2]. Therefore, understanding the impact of specific subsets of immune cells that infiltrate tumours is important for making rational decisions in the development of targeted therapies.

Oesophageal adenocarcinoma (OAC) is becoming increasingly common in the Western world, and despite a variety of strategies to improve outcome [5], survival remains poor at 10–15 % at 5 years [6]. Radical treatment with curative intent includes neoadjuvant therapy and oesophagogastrectomy. Neoadjuvant chemotherapy (NAC) delivers improved survival in a small percentage of patients, in whose tumours there is a significant pathological response to treatment [7]. However, approximately two-third of patients present with advanced, incurable disease at diagnosis. Biological therapies such as growth factor blockers, poly-ADP ribose polymerase (PARP) inhibitors, vaccines and monoclonal antibodies are being evaluated and are yet to enter routine clinical practice [8]. The rapid rise in incidence of OAC in the west means that novel therapies are urgently required, either as single agents or for use in combination with conventional treatments.

In particular, the ability of CD8+ lymphocytes to recognise tumour antigens has been well documented [9], and tumour regression is observed when tumour-reactive T lymphocytes invade cancers [10]. Before considering immune therapies as a potential treatment in OAC, it is important to understand whether immune cells are present in OAC, in which subsets of cells are observed and whether or not they have prognostic significance.

The effects of TILs have been studied in a range of solid tumours, and high TIL density correlates with better survival (reviewed in [11]). A few initial studies have examined the association of TILs in OAC with prognosis [12, 13]. These studies have provided conflicting results, and no study has assessed TIL density in resected tumours after NAC and whether or not there is a link to outcome. Schumacher et al. observed intratumoural CD8+ T cell infiltration to correlate with improved survival in a small heterogeneous cohort of OAC (*n* = 37) and oesophageal squamous cell carcinoma (OSCC) (*n* = 33) patients [14]. Additional studies have shown that the overall grade of TIL density at the invasive margin correlates with improved survival in patients with gastroesophageal cancer [12]. In contrast, Zingg et al. did not find any independent associations between differing TIL subsets and survival in OAC patients who received multimodal therapy with either neoadjuvant chemoradiotherapy or surgery alone. However, they did demonstrate in univariate analyses that particular subtypes of TIL conferred a better survival when dichotomised at median counts (CD3+, CD8+, FOXp3+, CD8+:CD4+) [15]. Therefore, the prognostic value of TILs in OAC remains to be established and the composition of TIL density following NAC in OAC is yet to be assessed.

In this study, we assessed the immune infiltrate in a large contemporary cohort of OAC (*n* > 120) to identify association with survival and clinicopathological disease characteristics. Specifically, we examined TILs, as their presence would suggest that active immunotherapy might be attractive in this condition. A link between pre-existing immunity might then also allow us to stratify patients into groups, more or less likely to benefit from immunomodulation such as checkpoint blockade, which allows a release of pre-existing immune responses for clinical benefit. In addition, we analyse TIL frequencies following NAC and assess the relationship between TILs and disease outcome.

## Materials and methods

### Patient selection

A prospectively collected database of consecutive patients undergoing oesophagogastric resection for OAC treated at University Hospital Southampton NHS Foundation Trust between January 2005 and December 2010 was reviewed. Patients were excluded from the study if they had achieved a complete pathological response (tumour regression grade (TRG) 1; no residual tumour), if they died post-operatively as an inpatient, or if no histopathological tissue was available. Ethical approval was received from the Southampton and South West Hampshire Research and Ethics Committee (REC 09/H0504/66) and approved by the local research and development department (RHM CAN0649).

### Patient clinical, pathological, treatment and follow-up characteristics

All patients (*n* = 128, Table 1) were discussed at a specialist multidisciplinary team meeting. Standard staging investigations included endoscopic ultrasonography, high-resolution computed tomography, integrated fluorodeoxyglucose positron emission tomography/computed tomography and staging laparoscopy, where indicated and were uniformly applied during the study interval. Patients considered suitable for potential surgical resection with tumours staged as T2 N0 M0 or above were considered for NAC.

**Table 1:** Clinicopathological characteristics of patient cohort for which formed TMAs

	*n* = 128
Operation age*	67.77 (45.48–85.41)
Sex ratio (M:F)	112:16
ASA	
1	13 (10.2)
2	93 (72.4)
3	22 (17.3)
Tumour site	
Lower 1/3	46 (35.9)
OGJ—S1	25 (19.5)
OGJ—S2	26 (20.3)
OGJ—S3	31 (24.2)
Type	
AC	128 (100)
pT or ypT	
T1	29 (22.7)
T2	30 (23.4)
T3	65 (50.8)
T4	4 (3.1)
pN or ypN	
N0	59 (46.1)
N1	28 (21.9)
N2	21 (16.4)
N3	20 (15.6)
pM or ypM	
M0	125 (97.7)
M1	3 (2.3)
Resection clearance (R0)	104 (81.3)
Vascular invasion	51 (39.8)
Lymphatic invasion	21 (16.4)
Perineural invasion	17 (13.3)
Neoadjuvant chemotherapy	76 (59.4)
Neoadjuvant regime	
ECX	64 (50)
EOX	10 (7.8)
ECF	2 (1.6)
Tumour regression grade	
1	0 (0)
2	11 (8.6)
3	10 (7.8)
4	29 (22.7)
5	26 (20.3)
Not assessed	0 (0)
Surgery only	52 (40.6)
Nodal downstaging	30/76 (39.5)

Values in parentheses are percentages unless indicated

ASA American Society of Anesthesiologists physical status classification system

* Values in parentheses are range

NAC consisted of three 21-day cycles of anthracycline, platinum and fluoropyrimidine: ECF (epirubicin 50 mg/m^2^, cisplatin 60 mg/m2, both intravenously on day 1 and venous infusion 5-FU 200 mg/m2 per day for 21 days), ECX (epirubicin 50 mg/m2, cisplatin 60 mg/m2, both intravenously on day 1 and capecitabine 625 mg/m2 orally twice daily for 21 days) or EOX (epirubicin 50 mg/m2 i.v. bolus and oxaliplatin 130 mg/m2 i.v. infusion over 2 h on day 1, capecitabine 625 mg/m2 orally twice daily for 21 days).

Pathological status after chemotherapy was assessed using the TRG system developed by Mandard et al. [16] who scored regression based on the degree of fibrosis and number of residual cancer cells (TRG 1–5). TRG was scored by specialist gastrointestinal pathologists, initially by one pathologist (Adrian C Bateman) prior to its introduction by all pathologists as part of routine pathological reporting. All dissected lymph nodes were stained with haematoxylin and eosin and microscopically analysed for metastatic disease. Lymph node downstaging was defined by lymph nodes being positive for signs of cancer (cN+) at diagnosis, assessed radiologically (computed tomography, positron emission tomography, endoscopic ultrasonography) and then pathologically recorded as the lymph nodes showing no signs of cancer spread (ypN0) after NAC given prior to surgery as previously described [17].

Surgery was performed after initial staging or 4–6 weeks following NAC as previously described [18].

Data recorded included demographics, tumour characteristics, resection type and histopathological analysis of the surgical specimen. The TNM classification of malignant tumours (TNM) 7 was used to report tumour stage after analysis of pathology reports [19]. Pathological tumour clearance (“R”-status) was determined according to the Royal College of Pathologists’ guidance [20].

All patients were cared for by a specialist oesophagogastric team as previously described [18]. Recurrence of disease during follow-up was defined as the first site or sites of recurrence with radiological or pathological confirmation. Cancer-specific survival (CSS) was defined as time of operation to death in the absence of other causes of death. For assessment of disease-free survival (DFS), recurrence was defined as time from operation to development of local, nodal (regional) and distant metastasis (whichever occurred first).

### Immunohistochemistry

Post-operative tumour histology was reviewed by pathologists (Gareth J. Thomas and Adrian C. Bateman) blinded to treatment and outcome, and a suitable paraffin block was selected. Tissue microarrays (TMAs) were constructed using triplicate, randomly selected, paraffin-embedded 1-mm tumour cores (Aphelys Minicore 2, Mitogen, Harpenden, UK). To assess the immune infiltrate, 4-µm sections of TMA blocks were used. Automated immunostaining (Ventana XT, Ventana, Tucson, AZ, USA) was performed in a clinical pathology accredited cellular pathology department using antibodies optimised to national diagnostic standards (National External Quality Assessment Service). Antibodies to assess antigens were as follows: CD3, pan T cells; CD4, helper T cells; CD8, cytotoxic T cells (all from Novocastra, Milton Keynes, UK); and FOXp3, regulatory T cells (eBioscience, Hatfield, UK). Patients (*n* = 13) with complete pathological response (TRG 1) were excluded due to the inability to assess TILs due to the lack of tumour.

### Immunohistochemical evaluation

All sections were digitally image captured to enable sections to be scored under the supervision of an experienced pathologist (Gareth J. Thomas). Three high-powered fields (×400 magnification) with the highest density of the marker of interest were scored, with a mean taken, providing a total scoring area of 0.1944 mm2. This is in keeping with previous studies assessing intratumoural immune infiltration [21].

Training of the observers was undertaken on a series of OACs from the TMA. A total of 25 randomly selected patients from the total cohort were used to assess the intraobserver and interobserver variation in the TIL scoring. Two observers (Fergus Noble and Leo M. Matthews) assessed the TIL infiltrate independently and without knowledge of clinicopathological information. The interobservers’ intraclass correlation coefficient was >0.7 and was considered acceptable in keeping with previous studies [22]. Fergus Noble scored all slides, and these data were used in the analysis [23].

Following scoring, TIL density data were grouped to facilitate some of the statistical analysis. This was established using justifiable cut-offs (high–low/either side of the median).

### Preoperative systemic inflammatory and nutritional blood-borne markers analysed

Preoperative blood samples were taken for routine laboratory analysis of full blood count (FBC) and albumin in the preoperative period (within 1 week of resection). The white cell count (WCC) (reference range 4.0–11.0 × 109/l), platelet count (reference range 150–400 × 109/l), neutrophil count (reference range 2.0–7.5 × 109/l) and lymphocyte count (reference range 1.5–4.0 × 109/l) were analysed with an automated haematological blood analyser (Sysmex TS-500 (Sysmex UK Ltd)). Serum concentrations of albumin (normal range: 35–48 g/l) were measured in an auto-analyser (UniCel DxC800 (Beckmann Coulter Inc)). The coefficient of variation for these methods, over the range of measurement, was less than 2 % as established by routine quality control. All patients were free from infection at the time of blood collection as determined by clinical assessment.

The neutrophil/lymphocyte ratio (NLR) was calculated by dividing the absolute neutrophil count by the absolute lymphocyte count (reference range 0.5–5) and represents an inexpensive measure of systemic inflammation [24].

### Statistical analysis

Descriptive data are represented as median and range unless indicated. The Kruskal–Wallis, Mann–Whitney U and Pearson’s Chi-squared tests were used as appropriate for comparisons of groups. Kaplan–Meier, univariate and multivariate Cox logistic regression modelling were used to assess the relationship between immune infiltration with CSS, DFS and response to NAC as outcomes. All factors that showed statistical significance on univariate analysis were entered to derive the final model using the backward stepwise likelihood ratio method. CSS and DFS curves of the patients were plotted by using the Kaplan–Meier method and analysed using the log-rank test. Stratified analyses were performed based on receipt of NAC and pathological response to chemotherapy. A* p* value < 0.05 was considered statistically significant for all tests. Statistical analysis was performed with statistical package for the social sciences version 21 (SPSS®, Chicago, Illinois, USA).

## Results

### Study patients

A total of 128 patients were included in the study with a median follow-up of 3.5 years (95 % CI 2.629–4.342). The study population had a median age at operation of 68 years (range, 46–85 years) and was predominately male (88 %). The majority had pathologically advanced staged, pT/ypT3 (51 %) and node-positive disease (54 %), which were located either at the oesophagogastric junction (64 %) or in the lower oesophagus (36 %).

In total, 76 (59.4 %) patients received multimodal therapy, consisting of NAC and surgery, with 52 (40.6 %) patients proceeding directly to surgery alone. Of the patients who received multimodal therapy, 11 (15 %) patients had a significant local tumoural response (TRG 2), and 30 (40 %) patients had a significant lymph node response (lymph node downstaging: cN+ to ypN0) to NAC.

Detailed patient characteristics and clinical and pathological outcomes are summarised in Table 1.

### The relationship of intratumoural infiltrating lymphocytes and clinicopathological characteristics

Representative TIL staining is shown in supplemental Figure S1. The relationship of patient and tumour characteristics to TILs is presented in supplemental Table S1 and supplemental Table S2, respectively.

A lower TIL density, across all subsets, was significantly associated with increasing stage of disease (T and N stage) and with vascular, lymphatic and perineural invasion (supplemental Table S2).

Higher TIL counts were observed in female compared to male patients (supplemental Table S1). This was statistically significant for CD4+ (*p* = 0.036), CD8+ (*p* = 0.037) and FOXp3+ (*p* = 0.045) cells. No statistically significant differences were found regarding premorbid status (performance status, ASA grade, age or smoking status, supplemental Table S1).

**Fig. 1:**
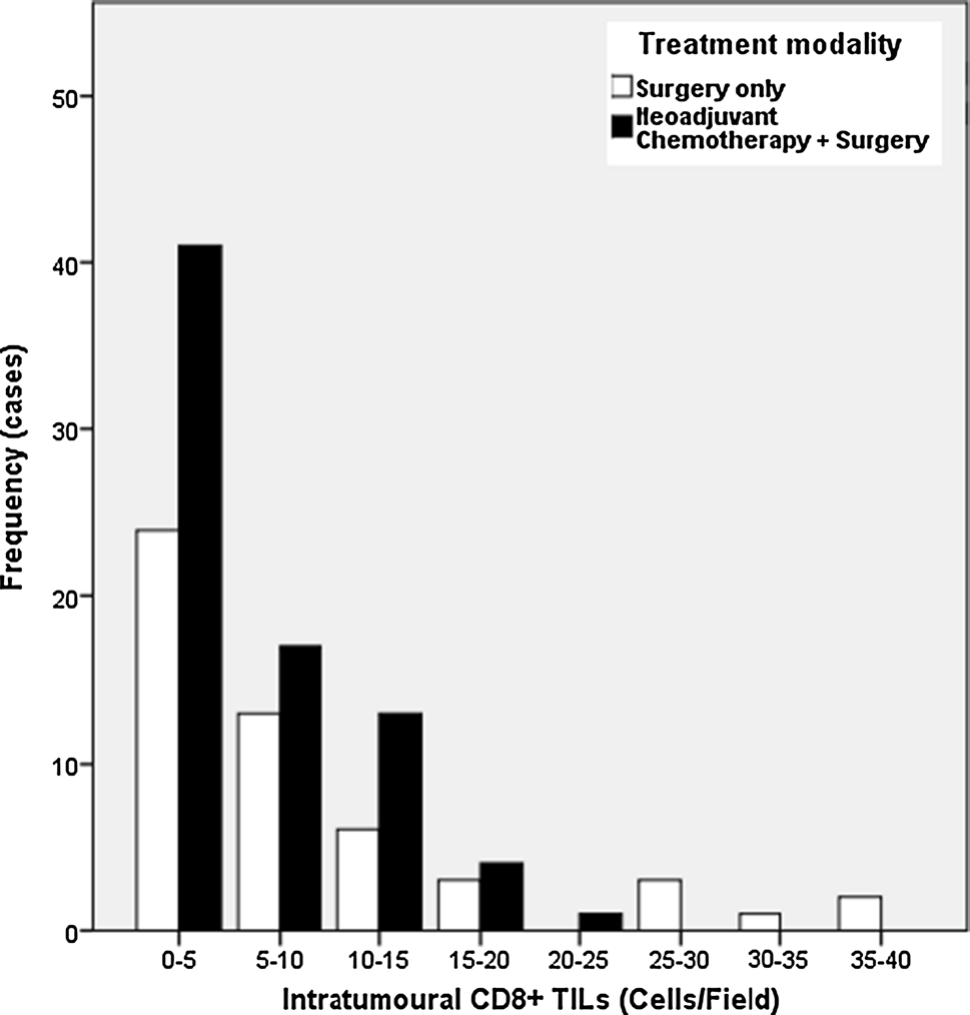
Frequency distribution of CD8+ TILs by treatment modality highlighting the low proportion of patients with high TIL infiltration

The relationship of TILs and outcome was not statistically different between patients treated with multimodal therapy, NAC and surgery, or surgery alone and is presented in supplemental Table S1 and supplemental Table S3. The distribution of TILs was positively skewed with a heavier left than right tail indicating a low proportion of patients have high levels of infiltration with lymphocytes. The distribution of CD8+ TILs is shown as an example in Fig. 1.

The number of TILs positively correlated with each other for all subtypes (range of correlation coefficient = 0.677–0.905; *p* < 0.001) and is detailed in supplemental Table S4.

### Prognostic significance of intratumoural infiltrating lymphocytes

**Fig. 2:**
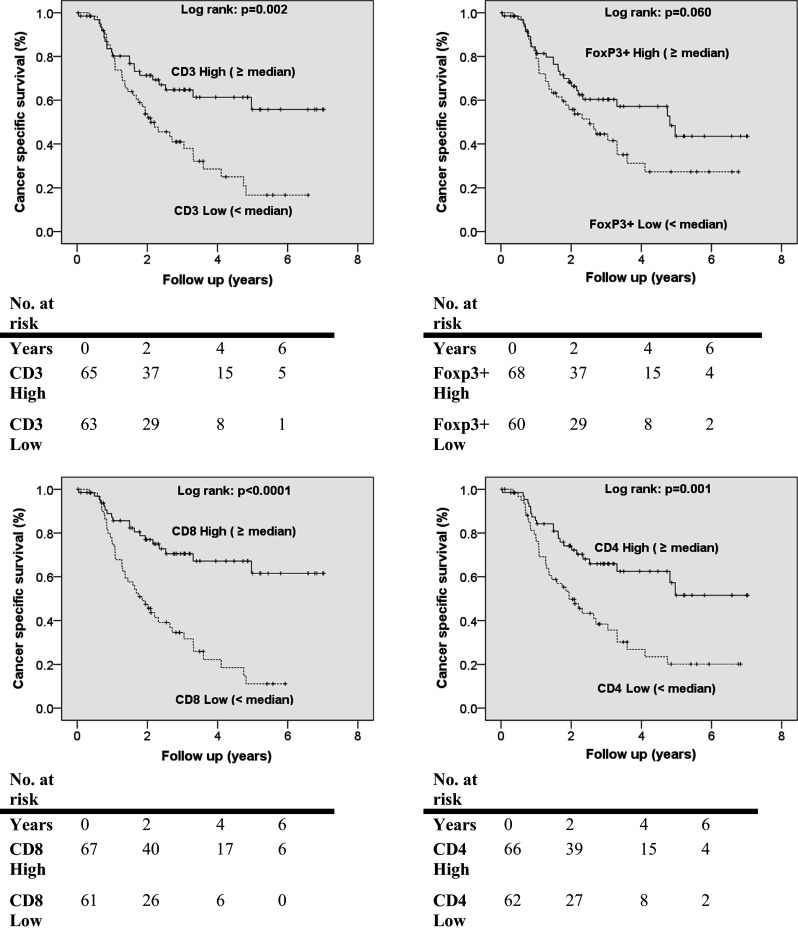
Kaplan–Meier curves of cancer-associated survival revealing prognostic significance of TILs in oesophageal adenocarcinoma

Median follow-up was 3.5 years with the results of the Cox proportional hazard model for predictors of CSS and DFS shown in Table 2 and supplemental Table S5, respectively. Multivariate analysis identified independent prognostic factors for improved CSS to be reduced pathological N stage (*p* < 0.0001), higher CD8+ TILs (*p* = 0.003) and completeness of resection (*p* < 0.0001). The hazard ratio for higher number of CD8+ TILs was 0.847 (95 % CI 0.760–0.944). Segregation of the TIL data either at the medians (low < median or high ≥ median TIL levels) allowed Kaplan–Meier survival analysis; the data presented in Fig. 2 demonstrate that higher numbers were associated with improved CSS. CD8+ TILs ≥ 5 were associated with better CSS [CD8+ TILs ≥ 5: mean (median not yet reached) CSS 5.1 years, 95 % CI 4.4–5.8, vs. CD8+ TILs < 5: median CSS 1.9 years, 95 % CI 1.3–2.4, *p* < 0.0001].

**Table 2:** Univariate and multivariate Cox regression analyses of patient and tumour factors with OAC-specific survival

	Univariate			Multivariate		
	HR	95 % CI	*p* value	HR	95 % CI	*p* value
Patient factors						
Age	0.987	0.961–1.014	0.348			
Sex						
Female	1	Ref				
Male	1.088	0.495–2.389	0.834			
ASA						
1	1	Ref				
2	1.670	0.659–4.231	0.280			
3	1.398	0.485–4.031	0.535			
Performance status						
0	1	Ref				
1	0.867	0.475–1.580	0.640			
2	0.606	0.199–1.843	0.377			
Preoperative smoker						
No	1	Ref				
Yes	0.835	0.491–1.421	0.507			
Neoadjuvant Rx						
No	1	Ref		1	Ref	
Yes	1.723	1.011–2.937	**0.046**	1.703	0.977–2.971	0.061
Immunohistochemistry						
CD3	0.966	0.947–0.985	**0.001**	1.035	0.997–1.075	0.075
CD4	0.927	0.874–0.982	**0.010**			
CD8	0.895	0.849–0.944	**<0.0001**	0.847	0.760–0.944	**0.003**
FOXp3+	0.879	0.801–0.966	**0.007**			
Tumour factors						
ypT or pT stage						
1	1	Ref				
2	2.450	0.947–6.338	0.065			
3	4.480	1.872–10.720	**0.001**			
4	16.094	4.385–59.071	**<0.0001**			
ypN or pN stage						
0	1	Ref		1	Ref	
1	4.036	2.033–8.014	**<0.0001**	3.745	1.806–7.764	**<0.0001**
2	6.063	2.889–12.723	**<0.0001**	3.476	1.515–7.974	**0.003**
3	5.143	2.464–10.735	**<0.0001**	3.607	1.635–7.959	**0.001**
ypM or pM stage						
0	1	Ref				
1	4.546	1.402–14.746	**0.012**			
Vascular invasion						
No	1	Ref				
Yes	2.234	1.354–3.684	**0.002**			
Lymphatic invasion						
No	1	Ref				
Yes	1.985	1.109–3.554	**0.021**			
Perineural invasion						
No	1	Ref				
Yes	2.811	1.519–5.201	**0.001**			
Resection clearance						
R0	1	Ref		1	Ref	
R1	3.027	1.737–5.273	**<0.0001**	3.134	1.699–5.783	**<0.0001**

Bold values indicate variables of significance

Multivariate analysis identified independent prognostic factors for reduced DFS as lower CD8+ TIL numbers (*p* < 0.0001), pathological N stage (*p* < 0.0001), incompleteness of resection (*p* = 0.001) and multimodal treatment (*p* = 0.015). The hazard ratio for higher number of CD8+ TILs was 0.894 (95 % CI 0.844–0.948). Kaplan–Meier survival analysis is presented in supplemental Figure S2 showing that higher levels of TILs were associated with improved DFS. CD8+ TILs ≥ 5 were associated with better DFS (CD8+ TILs ≥ 5: mean (median not yet reached) DFS 4.7 years, 95 % CI 3.9–5.4, vs. CD8+ TILs < 5: median DFS 1.2 years, 95 % CI 0.9–1.5, *p* < 0.0001).

### The relationship of intratumoural infiltrating lymphocytes and response to NAC

Significantly higher TIL numbers were found in the tumours of patients who had a better pathological response to NAC, both when assessing response based on the tumour (TRG) and the lymph nodes (downstaging of lymph nodes from pretreatment clinical staging to pathological N stage), as detailed in Tables 3, 4, supplemental Table S6, supplemental Table S7 and supplemental Figure S3. There was a higher CD3+ (*p* = 0.007), CD4+ (*p* = 0.025) and CD8+ count (*p* = 0.002) in the tumour of patients whose tumour nodal status was downstaged at pathological evaluation after NAC. Higher TIL frequencies were also seen in patients who had a significant pathological response to NAC (TRG2) compared to those with no significant response (TRG 3–5) (Table 3).

**Table 3:** Clinical and pathological factors in OAC patients that received chemotherapy (*n* = 76) based on their response to neoadjuvant chemotherapy (LN downstaged)

	Responder *n* = 30	Non-responder *n* = 46	*p* value
Age*	63.68 (45.48–77.75)	62.62 (50.67–81.28)	0.941
Sex			
Male	29 (96.7)	39 (84.8)	0.101
Female	1 (3.3)	7 (15.2)	
Performance status			
0	8 (26.7)	14 (30.4)	0.446
1	20 (66.7)	32 (69.6)	
2	2 (6.7)	0 (0)	
ASA			
1	5 (16.7)	5 (11.1)	0.859
2	20 (66.7)	37 (80.0)	
3	5 (16.7)	4 (8.9)	
ypT			
1	11 (36.7)	2 (4.3)	**<0.0001**
2	10 (33.3)	11 (23.9)	
3	9 (30.0)	31 (67.4	
4	0 (0)	2 (4.3)	
ypN			
0	30 (100)	3 (6.5)	**<0.0001**
1	0 (0)	15 (32.6)	
2	0 (0)	15 (32.6)	
3	0 (0)	13 (28.3)	
Tumour response			
Yes (TRG2)	11 (36.7)	0 (0)	**<0.0001**
No (TRG 3–5)	19 (63.3)	46 (100)	
Differentiation			
G1—well	5 (16.7)	37 (80.4)	**0.036**
G2—Moderate	9 (30.0)	1 (2.2)	
G3—poor	16 (53.3)	11 (23.9)	
Resection clearance			
R0	2 (6.7)	12 (26.1)	**0.034**
R1	28 (93.3)	34 (73.9)	
Immunohistochemistry values*			
CD3	15.15 (0.00–44.30)	11.20 (0.00–64.00)	**0.007**
CD4	5.00 (0.00–27.00)	2.30 (0.00–20.00)	**0.025**
CD8	8.65 (0.00–20.70)	4.50 (0.00–18.70)	**0.002**
FOXp3+	1.85 (0.00–11.30)	1.00 (0.00–9.30)	**0.074**

Values in parentheses are percentages unless indicated

* Values in parentheses are range

Bold values indicate variables of significance

**Table 4:** Univariate and multivariate analysis of immunohistochemical markers for response to neoadjuvant chemotherapy (LN downstaged) in OAC

	Univariate			Multivariate		
	HR	95 % CI	*p* value	HR	95 % CI	*p* value
CD3	0.957	0.924–0.992	**0.015**			
CD4	0.916	0.838–1.001	0.052			
CD8	0.869	0.789–0.958	**0.005**	0.869	0.789–0.958	**0.005**
FOXp3+	0.868	0.734–1.026	0.097			

Bold values indicate variables of significance

On multivariate analysis higher CD4+ TIL (*p* = 0.017) and CD8+ TIL densities (*p* = 0.005) were associated with significant tumour response (TRG) and lymph node downstaging, respectively, shown in supplemental Table S7 and Table 4.

### The relationship of intratumoural infiltrating lymphocytes and preoperative systemic inflammatory markers

Higher TIL numbers were found in the tumours of patients with normal serum albumin although this did not reach statistical significance [albumin < 35: CD8+ numbers 2.00 (0.00–29.30) vs. albumin ≥ 35: CD8+ numbers 6.00 (0.00–38.00), *p* = 0.069 (supplemental Table 1)].

## Discussion

In this study we have analysed the level of TILs in a large homogeneous cohort of oesophagogastric cancer patients following radical treatment with curative intent. We obtained three major findings: firstly, we have shown associations between pathological stage of disease and TIL density and confirmed the independent association of particular TIL subsets and survival; secondly, we have shown significant correlation between TIL subtypes in OAC, and finally, we found patients with a significantly increased pathological response to NAC had higher levels of TILs in their resected tumour, most notably with CD4+ and CD8+ TILs for local tumour regression and lymph node response, respectively.

Of the TIL subsets analysed, CD8+ T cells had the most significant independent association for both CSS (*p* = 0.003) and DFS (*p* < 0.0001). This has been previously suggested to be important in OAC; however, independent association has not been universally verified [12, 14, 15]. Zingg el al. [15] identified on univariate analysis a number of TILs as significant predictive factors; however, these were not independent factors when taking into account the stage of disease. In contrast, Schumacher et al. [14] identified CD8+ TILs to be a significant predictive factor of survival independent of disease stage. The reasons for this discrepancy of findings between studies may relate to the clinicopathological factors used to build the multivariate statistical model, the location of TILs counted and the cut-off values used to dichotomise the TIL frequencies into high and low groups. In our large and homogeneous cohort, we choose to evaluate TIL number as a continuous variable, counted intratumoural TILs and entered a comprehensive number of known clinicopathological predictors of survival into the univariate and subsequent multivariate statistical models. We believe this to be the most robust analysis of the association of TILs with survival on OAC that has been conducted to date.

In addition to the observed effects TILs have on disease recurrence, this study demonstrates that TILs play a role at different stages of disease. The significant association observed between TILs with T and N stage supports the possibility that intratumoural T cells prevent tumour progression throughout the disease process. These findings are supported by previous smaller studies showing correlation with low TILs and higher stage of disease [14, 15].

A novel observation is that the response to chemotherapy links with TIL counts. Previous studies have highlighted increased infiltrate of TILs in OAC tumours after NAC when compared to surgery alone [13]. We did not find any statistically significant difference between TIL levels in those patients who received NAC and those that proceeded directly to surgery as a whole. It would be preferable to evaluate TILs in preoperative biopsies to assess response prior to neoadjuvant therapy. This was attempted but was unsuccessful due to the paucity of tumour material in the small diagnostic endoscopic biopsies unlike the diagnostic biopsies taken for other tumours that tend to be larger, for example breast and colon cancer. However, different and heterogeneous chemotherapy regimens were used in previous studies making comparison difficult. We did find that patients who had a significant response to NAC (as assessed by TRG and LN response) had a higher level of immune infiltrate. This important finding has been shown in other cancer sites where loss of CD4+ Treg [23] and TIL numbers at the margins of liver metastases [24] predicted for response to chemotherapy [25–27]. The potential immunological mechanisms by which cytotoxic chemotherapy can provide anti-tumour activity are being increasingly highlighted. These include: subverting immunosuppressive mechanisms; exerting stimulatory effects in immune cells; and modulating dying tumour cells, so they regain visibility to the host immune response [28]. The combination of immunological therapies with conventional chemotherapy has been suggested to provide a synergistic effect if the host immunological response is harnessed appropriately [29]. In addition to the effects of chemotherapy on TILs, previous studies have highlighted spontaneous pathological regression of tumours in 13.7 % (*n* = 17/124) of OAC cases that have received no NAC when using TRG to assess the resected tumour specimen. This may represent the host immunological response and successful immune attack [30]. Our study would support this hypothesis as significant response to NAC was associated with increased TILs most notably with CD8+ TILs. In other tumour sites, CD8+ infiltration has been shown to correlate with specific immunogenic antigen expression and improved survival [31]. These findings highlight the T cell effector potential and a means to harness the patient’s immune response in cancer.

It is also of interest that increased frequencies of TILs correlate with improved response following the use of trastuzumab [32] in light of the ToGA study [33] that may lead to the adjuvant use of trastuzumab in OAC. This adjuvant therapy may lead to a further increase in the adaptive immune response in the tumour as seen in breast cancer [34]. We have previously shown that patients are more likely to respond to chemotherapy if acute phase proteins are normal (serum albumin) by assessing systemic markers of nutrition and the inflammatory response (neutrophil/lymphocyte ratio, serum albumin) [18]. It is suggested that suboptimal immunological and nutritional status may contribute to tumour development through subversion of tumour immunity [35, 36] and this is particularly pertinent to OAC. We found that patients with lower TIL levels were less likely to respond to chemotherapy and had lower serum albumin levels. From our data, it is not possible to know whether high TIL levels lead to a greater responsiveness to chemotherapy or chemotherapy leads to a higher number of TILs in those that respond, and it may well be a combination of the two. The association of poor nutritional status with lower TIL levels and a lack of response are logical and may well be the most likely explanation.

Limitations of this study include its retrospective nature and the associated biases on selection and collection. Patients were excluded if they had a complete pathological response to NAC due to the lack of tumour in the resection specimen and also if there was insufficient material collected. However, this cohort is representative of current clinical practice. We found increasing nodal burden (*p* < 0.0001) to be the best independent prognosticator for worse survival. The cohort was homogenous in terms of staging, histology and treatment algorithms. In addition, patient, tumour factors and survival data are in keeping with published western cohorts making our findings applicable to these populations. The excluded patients with complete pathological response (TRG1) potentially will have had the highest immune response with high TIL levels. This hypothesis is supported by analysis of the cytotoxic response of patients with a complete pathological response in breast cancer patients. Granzyme B and TiA1, cytolytic granules, expressing cells were observed at higher frequency in specimens that had undergone a pathological complete response [25].

Additional criticism could be expressed with regard to the use of TMA cores not being representative of the tumour as a whole. However, multiple studies have utilised this approach and excellent correlation between the two has been established [37]. With respect to scoring TILs, the technique used has been described frequently by other studies with good interobserver and intraobserver correlation. Furthermore, these data were analysed with and without dichotomization so as to limit false-positive results [38, 39].

An unexpected finding in our study was the association of higher TILs for some subsets (CD4+, CD8+ and FOXp3+) in female patients. This may represent a type 1 error; however, it is well documented that immune cell numbers vary with age and gender. Possible explanations include the inhibitory effect of oestrogen on T-suppressor cells or its stimulatory effect on T helper cells [40]. However, this speculation is outside the remit of this study and will require further analysis. The reason why higher infiltration of regulatory FOXp3+ cells showed favourable prognosis in this study is complex but has been reported in gastric and other tumour types [41, 42]. This warrants further functional analysis, but others have suggested a synergistic role of regulatory cells with other TIL subsets within the tumour [43].

With regard to our novel finding, it remains to be seen whether the composition of TILs can predict for response to chemotherapy prior to treatment and the functional role the immune response plays in improving the response to chemotherapy. Emerging evidence from other solid organ tumours suggests that this might be possible [44]. However, as yet the required sensitivity and specificity have not been met for this to enter clinical practice. A potential clinical application of the TIL response could be in the selection of patients for specific adjuvant therapies, specifically using immunomodulators. In our series, a significant response to chemotherapy (TRG2 or LN downstaging) was associated with high TIL levels, suggesting these patients may benefit from further adjuvant chemotherapy. It would be intriguing in this group in particular to evaluate the long-term outcome after the use of immunomodulators, that release pre-existing anti-tumour T cells, as has been proposed for aPD1 and aCTLA4 therapies. In contrast, the group of patients who had no or a partial response to chemotherapy (TRG 3–5 or no LN downstaging) with high TIL levels in the pathological specimen additionally may benefit from further adjuvant or alternative chemotherapy in combination with an immunomodulator. In contrast, those patients with low TILs may warrant the use of second-line therapies but it seems less likely that immune attack will be successful, unless second-line therapy can overcome the barriers to immunological visibility of the cancer.

In summary, the results of this study show that local immune responses, in particular the adaptive immune response, are important independent predictors of CSS and DFS in patients with OAC undergoing radical treatment. These findings highlight the role of the adaptive immune response in preventing tumour recurrence in OAC. A generalised immune response was observed with high correlation between TIL subtypes and at all stages of disease. This is pertinent with regard to the design of therapies as it suggests all patients would benefit from treatment that appropriately boosts the immune response. A significant response was associated with higher infiltration with TILs post-therapy. Further work analysing the function of these TIL subsets and the correlation of pretreatment TIL densities with response to therapy may unearth predictors of response to chemotherapy in addition to aid design of novel treatments or as a mechanism to improve response to current therapies.

### Electronic supplementary material

Below is the link to the electronic supplementary material.

Supplementary material 1 (PDF 624 kb)

## Abbreviations

ASA: American Society of Anesthesiologists physical status classification system
CD: Cluster of differentiation
CSS: Cancer-specific survival
DFS: Disease-free survival
FOXp3: Forkhead box p3
LN: Lymph node
NAC: Neoadjuvant chemotherapy
NLR: Neutrophil/lymphocyte ratio
OAC: Oesophageal adenocarcinoma
OSCC: Oesophageal squamous cell carcinoma
PARP: Poly-ADP ribose polymerase inhibitors
TIL: Tumour infiltrating lymphocyte
TMA: Tissue microarray
TNM: The TNM classification of malignant tumours
TRG: Tumour regression grade
WCC: White cell count
