# Supplementary material for: Tumour infiltrating lymphocytes correlate with improved survival in patients with oesophageal adenocarcinoma
Source: Cancer Immunol Immunother. 2016 Mar 28;65(6):651–62. doi: 10.1007/s00262-016-1826-5 (PMC4880639; doi:10.1007/s00262-016-1826-5)
Supplement: Supplementary file 1 — Supplementary material 1 (PDF 624 kb) [file 262_2016_1826_MOESM1_ESM.pdf]

Supplemental Table S1 Relationship of TILs and OAC patient characteristics

		CD3+	CD4+	CD8+	FOXP3+
**Age/ years**	*p*-value	0.521	0.347	0.529	0.308
	**<75**	11.00 (0.00-27.00)	3.30 (0.00-27.00)	4.85 (0.00-37.70)	1.30 (0.00-21.00)
	**≥75**	14.00 (0.00-77.30)	5.00 (0.00-43.00)	5.85 (0.00-38.00)	2.00 (0.00-14.70)
**Sex**	*p*-value	0.059	**0.036**	**0.037**	**0.045**
	**Male**	11.00 (0.00-74.00)	**3.30 (0.00-27.00**	**4.70 (0.00-38.00)**	**1.15 (0.00-21.00)**
	**Female**	22.20 (0.00-77.30)	**7.85 (0.00-43.00)**	**9.00(0.00-25.70)**	**2.50 (0.00-10.70)**
**ASA**	*p*-value	0.338	0.553	0.697	0.173
	**1**	14.30 (0.00-43.30)	2.30 (0.00-22.00)	6.00 (0.00-16.70)	1.00 (0.00-21.00)
	**2**	12.65 (0.00-77.30)	4.30 (0.00-43.00)	5.50 (0.00-37.70)	2.00 (0.00-13.30)
	**3**	4.65 (0.00-77.30)	1.00 (0.00-16.70)	1.35 (0.00-38.00)	0.00 (0.00-11.30)
**Performance Status**	*p*-value	0.944	0.664	0.893	0.818
**0**		12.30 (0.00-71.00)	2.30 (0.00-22.00)	5.00 (0.00-29.00)	1.70 (0.00-21.00)
**1**		10.85 (0.00-77.30)	4.00 (0.00-19.30)	5.00 (0.00-38.00)	1.00 (0.00-13.30)
**2**		14.85 (0.00-72.00)	4.50 (0.00-27.00)	6.35 (0.00-32.70)	1.30 (0.00-21.00)
**Smoker Preop**	*p*-value	0.110	0.088	0.174	0.147
**Yes**		16.00 (0.00-74.00)	4.50 (0.00-27.00)	6.70 (0.00-38.00)	2.00 (0.00-21.00)
**No**		10.70 (0.00-72.00)	3.30 (0.00-22.00)	4.70 (0.00-32.70)	1.00 (0.00-13.30)
**Serum Albumin**	*p*-value	0.178	0.073	0.069	0.074
	<35	4.65 (0.00-71.00)	1.80 (0.00-20.00)	2.00 (0.00-29.30)	0.00 (0.00-12.70)
	≥35	12.65 (0.00-77.30)	4.30 (0.00-43.00)	6.00 (0.00-38.00)	1.30 (0.00-21.00)
**Serum Neutrophil Lymphocyte Ratio**	*p*-value	0.901	0.986	0.618	0.531
	<2.5	11.70 (0.00-74.00)	3.30 (0.00-22.00)	5.00 (0.00-38.00)	1.70 (0.00-14.70)
	≥2.5	12.50 (0.00-77.30)	3.85 (0.00-43.00)	5.85 (0.00-37.70)	1.00 (0.00-21.00)
**Treatment**	*p*-value	0.945	0.949	0.996	0.864
**Surgery only**		12.00 (0.00-77.30)	3.85 (0.00-43.00)	5.85 (0.00-38.00)	1.30 (0.00-21.00)
**Neoadjuvant Rx + Surgery**		12.00 (0.00-64.00)	3.70 (0.00-27.00)	5.00 (0.00-20.70)	1.30 (0.00-11.30)

Values median with range in parentheses

Supplemental Table S2 Relationship of TILs and OAC tumour characteristics

		CD3	CD4	CD8	FOXP3+
**ypT or pT**	*p*-value	**0.040**	**0.014**	**0.020**	**0.084**
	**T1**	**15.00 (0.00-77.30)**	**7.00 (0.00-43.00)**	**7.00 (0.00-25.70)**	**2.00 (0.00-21.00)**
	**T2**	**12.85 (0.00-72.00)**	**4.85 (0.00-27.00)**	**6.50 (0.00-37.70)**	**2.00 (0.00-13.30)**
	**T3**	**11.70 (0.00-74.00)**	**2.30 (0.00-20.00)**	**4.70 (0.00-38.00)**	**1.00 (0.00-14.70)**
	**T4**	**0.00 (0.00-12.00)**	**0.00 (0.00-4.70)**	**0.00 (0.00-3.70)**	**0.00 (0.00-6.70)**
**ypN or pN**	*p*-value	**<0.0001**	**<0.0001**	**<0.0001**	**0.008**
	**N0**	**16.00 (0.00-77.30)**	**6.00 (0.00-43.00)**	**8.30 (0.00-37.70)**	**2.00 (0.00-21.00)**
	**N1**	**13.35 (0.00-29.30)**	**3.65 (0.00-10.70)**	**5.20 (0.00-15.00)**	**1.65 (0.00-9.30)**
	**N2**	**0.00 (0.00-49.30)**	**0.00 (0.00-10.70)**	**0.00 (0.00-29.30)**	**0.00 (0.00-9.30)**
	**N3**	**7.85 (0.00-74.00)**	**2.00 (0.00-20.00)**	**2.00 (0.00-38.00)**	**0.00 (0.00-14.70)**
**Resection clearance**	*p*-value	**0.090**	**0.040**	0.088	**0.017**
	**R1**	**8.35 (0.00-64.00)**	**1.15 (0.00-20.00)**	1.50 (0.00-18.70)	**0.00 (0.00-10.00)**
	**R0**	**13.35 (0.00-77.30)**	**4.00 (0.0043.00)**	5.85 (0.00-38.00)	**1.70 (0.00-21.00)**
**Vascular Invasion**	*p*-value	**0.048**	**0.030**	**0.011**	**0.027**
	**Yes**	**10.70 (0.00-64.00)**	**1.70 (0.00-27.00)**	**4.30 (0.00-29.30)**	**1.00 (0.00-9.30)**
	**No**	**13.70 (0.00-77.30)**	**4.30 (0.00-43.00)**	**6.70 (0.00-38.00)**	**1.70 (0.00-9.30)**
**Lymphatic Invasion**	*p*-value	**0.022**	**0.038**	**0.037**	**0.182**
	**Yes**	**3.30 (0.00-43.40)**	**1.00 (0.00-27.00)**	**1.30 (0.00-15.00)**	**0.00 (0.00-9.30)**
	**No**	**13.70 (0.00-77.30)**	**4.30 (0.00-43.00)**	**5.70 (0.00-38.00)**	**1.30 (0.00-21.00)**
**Perineural Invasion**	*p*-value	**0.016**	**0.001**	**0.025**	**0.033**
	**Yes**	**0.00 (0.00-36.00)**	**0.00 (0.00-11.00)**	**0.00 (0.00-14.00)**	**0.00 (0.00-8.00)**
	**No**	**12.30 (0.00-77.30)**	**4.00 (0.00-43.00)**	**5.70 (0.00-38.00)**	**1.30 (0.00-21.00)**

Values median with range in parentheses

Supplemental Table S3 Descriptive statistics for number of TILs in OAC stratified by treatment modality

		CD3+ TILs	CD4+ TILs	CD8+ TILs	FOXP3+ TILs
**Surgery Only**	**Mean***	19.49	5.69	8.13	3.42
	**Median***	12.00	3.85	5.85	1.30
	**Variance**	525.20	55.87	105.10	23.13
	**Range***	0.0-77.3	0.0-43.0	0.0-38.0	0.0-21.0
	**Skewness**	1.224	2.771	1.572	1.689
**Neoadjuvant Rx + Surgery**	**Mean***	15.57	4.94	6.11	3.42
	**Median***	12.00	3.70	5.00	1.30
	**Variance**	206.54	31.98	28.95	7.91
	**Range***	0.0-64.0	0.0-27.0	0.0-20.7	0.0-11.3
	**Skewness**	0.877	1.715	0.679	1.291
**Total**	**Mean***	17.16	5.25	6.93	2.81
	**Median***	12.00	3.70	5.00	1.30
	**Variance**	336.61	41.46	60.30	14.21
	**Range***	0.0-77.3	0.0-43.0	0.0-38.0	0.0-21.0
	**Skewness**	1.329	2.428	1.790	1.900

*Data are cells counted per field; No statistically significant difference by treatment modality

Supplemental Table S4 Correlation between subtypes of TILs

		FOXP3+	CD8+	CD4+
**CD3+**	*Correlation coefficient*	**0.791**	**0.905**	**0.836**
	p-value	**<0.0001**	**<0.0001**	**<0.0001**
**CD4+**	*Correlation coefficient*	**0.677**	**0.710**	
	p-value	**<0.0001**	**<0.0001**	
**CD8+**	*Correlation coefficient*	**0.695**		
	p-value	**<0.0001**		

Supplemental Table S5 Univariate and multivariate Cox regression analyses of patient and tumour factors with OAC disease free survival

		Univariate			Multivariate		
		HR	95%CI	P value	HR	95% CI	*p* value
Patient factors							
Age		0.978	0.953-1.003	0.085			
Sex	Female	1	Ref				
	Male	1.305	0.596-2.855	0.505			
ASA	1	1	Ref				
	2	1.571	0.672-3.674	0.297			
	3	1.028	0.373-2.833	0.957			
Performance status	0	1	Ref				
	1	0.771	0.442-1.344	0.359			
	2	0.644	0.237-1.749	0.388			
Preoperative smoker	No	1	Ref				
	Yes	0.915	0.555-1.509	0.727			
Neoadjuvant Rx	No	1	Ref		1	Ref	
	Yes	1.996	1.183-3.367	**0.010**	1.998	1.145-3.487	**0.015**
**Immunohistochemistr**							
CD3		0.960	0.941-0.980	**<0.0001**			
CD4		0.895	0.842-0.951	**<0.0001**			
CD8		0.886	0.841-0.933	**<0.0001**	0.894	0.844-0.948	**<0.0001**
FOXP3+		0.885	0.811-0.965	**0.006**			
Tumour factors							
pT stage	1	1	Ref				
	2	4.325	1.610-11.618	0.004			
	3	6.180	2.424-15.758	**<0.0001**			
	4	15.913	4.178-60.604	**<0.0001**			
pN stage	0	1	Ref		1	Ref	
	1	5.796	2.988-11.243	**<0.0001**	5.178	2.630-10.196	**<0.0001**
	2	7.899	3.822-16.324	**<0.0001**	3.584	1.639-7.840	**0.001**
	3	6.351	3.036-13.288	**<0.0001**	5.142	2.353-11.235	**<0.0001**
pM stage	0	1	Ref				
	1	4.479	1.374-14.598	**0.013**			
Vascular invasion	No	1	Ref				
	Yes	2.671	1.657-4.307	**<0.0001**			
Lymphatic Invasion	No	1	Ref				
	Yes	2.099	1.213-3.635	**0.008**			
Perineural Invasion	No	1	Ref				
	Yes	2.450	1.309-4.584	**0.005**			
Resection clearance	R0	1	Ref		1	Ref	
	R1	2.563	1.492-4.405	**0.001**	2.600	1.448-4.666	**0.001**

Supplemental Table S6 Clinical and pathological factors in OAC patients that received neoadjuvant chemotherapy (n=76) based on their response to chemotherapy using tumour regression grading (TRG)

		**Responder (TRG 2)** n=11	**Non-responder (TRG 3-5)** n=65	***p* value**
**Age***		66.58 (55.77-74.62)	62.46 (45.48-81.29)	0.253
**Sex**	**Male**	11 (100)	57 (87.7)	0.222
	**Female**	0 (0)	8	
**Performance status**	**0**	4 (36.4)	18 (27.7)	0.488
	**1**	7 (63.6)	45 (69.2)	
	**2**	0 (0)	2 (3.1)	
**ASA**	**1**	1 (9.1)	9 (14.1)	0.922
	**2**	9 (81.8)	48 (73.4)	
	**3**	1 (9.1)	8 (12.5)	
**ypT**	**1**	6 (54.5)	7 (10.8)	**<0.0001**
	**2**	4 (36.4)	17 (26.2)	
	**3**	1 (9.1)	39 (60.0)	
	**4**	0 (0)	2 (3.1)	
**ypN**	**0**	11 (100)	22 (33.8)	**<0.0001**
	**1**	0 (0)	15 (23.1)	
	**2**	0 (0)	15 (23.1)	
	**3**	0 (0)	13 (20)	
**LN downstaged**	**Yes**	11 (100)	19 (29.2)	**<0.0001**
	**No**	0 (0)	46 (70.8)	
**Differentiation**	**G1 – Well**	3 (27.3)	3 (4.6)	0.061
	**G2 –**	3 (27.3)	17 (26.2)	
	**G3 – Poor**	5 (45.5)	45 (69.2)	
**Resection clearance**	**R0**	11 (100)	51 978.5)	0.090
	**R1**	0 (0)	65 (21.5)	
**Immunohistochemistry values***	**CD3**	16.00 (0.00-43.30)	12.00 (0.00-64.00)	0.160
	**CD4**	6.00 (0.00-27.00)	3.30 (0.00-20.00)	0.055
	**CD8**	8.30 (0.00-16.70)	4.70 (0.00-20.70)	0.065
	**FOXP3+**	2.70 (0.00-11.30)	1.30 (0.00-9.30)	0.202

Values in parentheses are percentages unless indicated. *Values in parentheses are range

Supplemental Table S7 Univariate and multivariate analysis of immunohistochemical markers for response to chemotherapy (TRG 2) in OAC

	**Univariate**			**Multivariate**		
	HR	95%CI	P value	HR	95% CI	*p* value
**CD3**	0.967	0.927-1.009	0.119			
**CD4**	**0.885**	**0.800-0.978**	**0.017**	**0.885**	**0.800-0.978**	**0.017**
**CD8**	0.912	0.814-1.023	0.116			
**FOXP3+**	0.831	0.678-1.017	0.073			

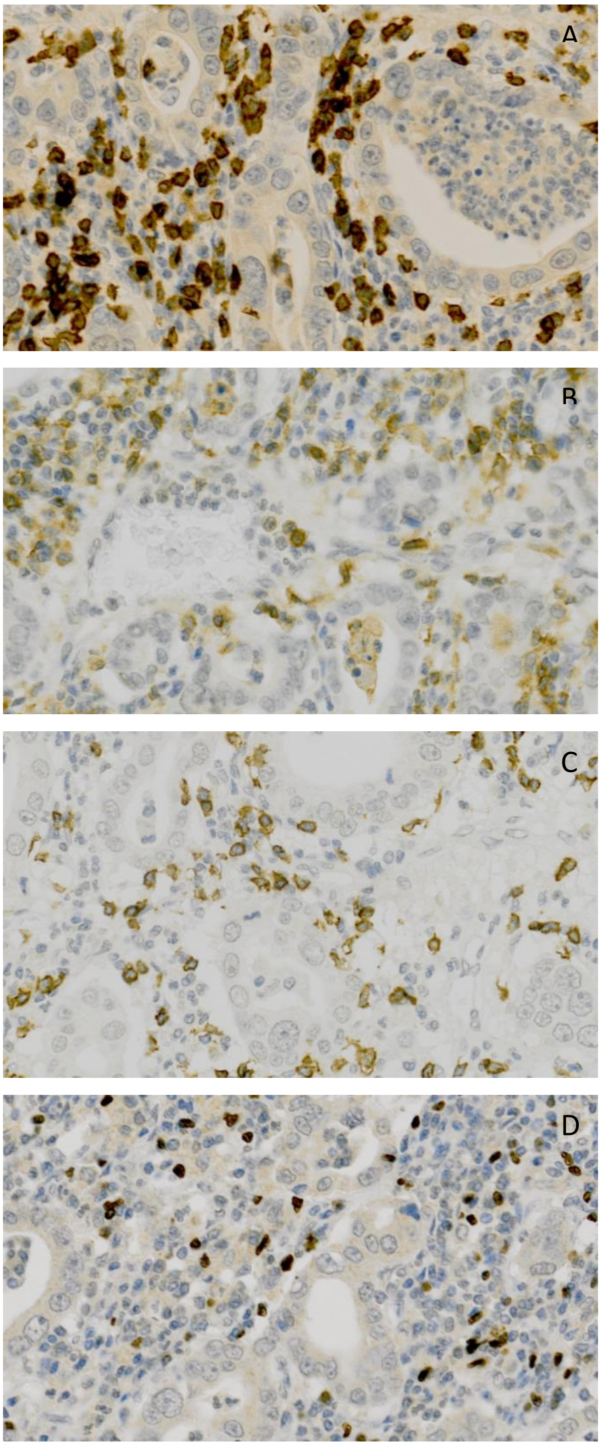

Supplemental Figure S1 OAC Tumour infiltrating lymphocytes CD3+ (A), CD4+ (B), CD8+ (C), FOXP3+ (D)

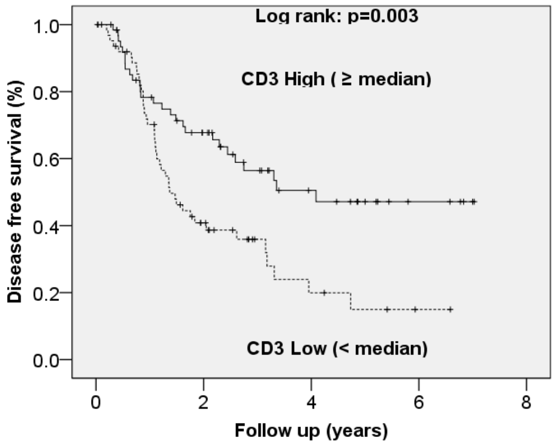

No. at risk

Years	0	2	4	6
CD3 High	65	35	15	5
CD3 Low	63	20	5	1

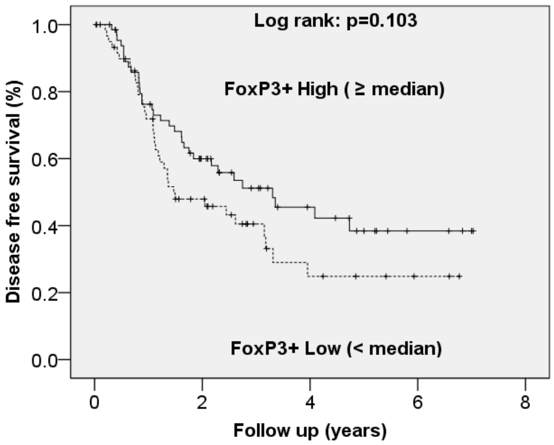

No. at risk

Years	0	2	4	6
FoxP3+ High	68	32	14	4
FoxP3+ Low	60	23	6	1

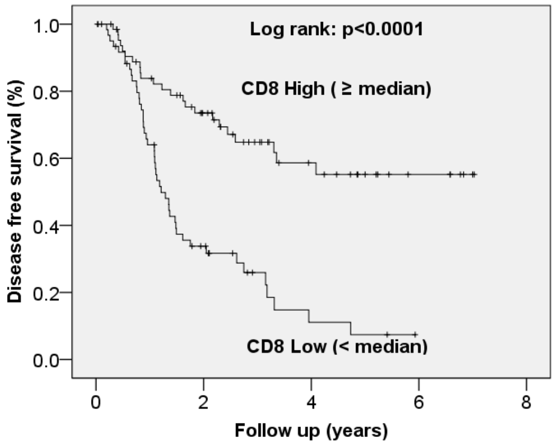

No. at risk

Years	0	2	4	6
CD8 High	67	38	17	6
CD8 Low	61	17	3	1

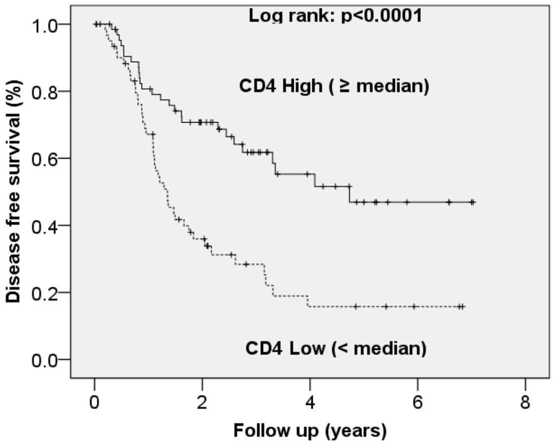

No. at risk

Years	0	2	4	6
CD4 High	66	37	15	4
CD4 Low	62	18	5	2

Supplemental Figure S2 Kaplan- Meier curves of disease free survival demonstrating prognostic significance of TILs in oesophageal adenocarcinoma

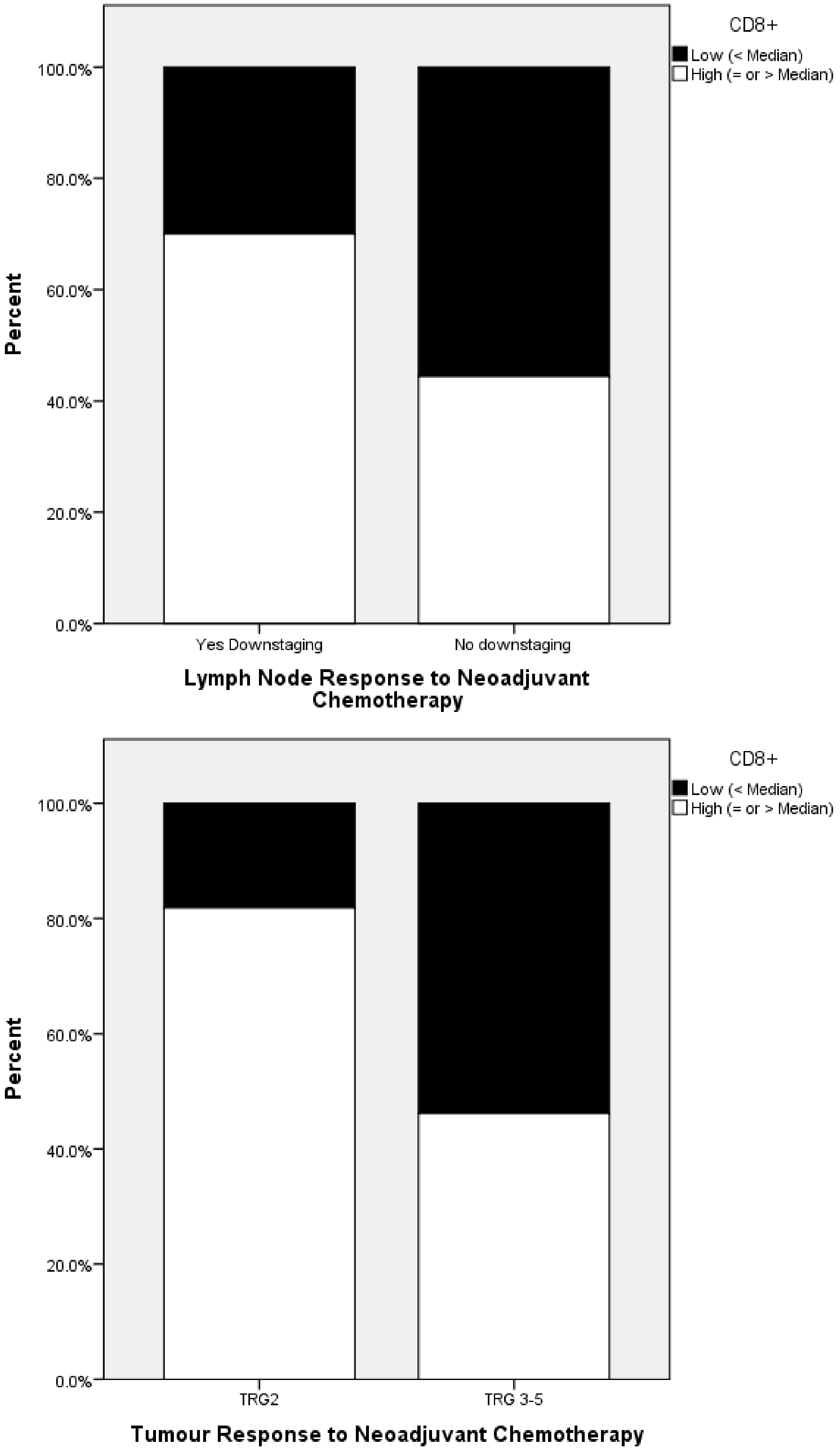

Supplemental Figure S3 CD8+ immune infiltrate in OAC assessed by tumour response to chemotherapy
